# Supplementary material for: Current use of attention-deficit hyperactivity disorder (ADHD) medications and clinical characteristics of child and adolescent psychiatric outpatients prescribed multiple ADHD medications in Japan
Source: PLoS One. 2021 Jun 3;16(6):e0252420. doi: 10.1371/journal.pone.0252420 (PMC8174687; doi:10.1371/journal.pone.0252420)
Supplement: Attachment — Submitted filename: Response to Reviewers_210417.docx [file pone.0252420.s001.docx]

**RESPONSE TO REVIEWERS**

**PONE-D-21-04661**

**Status of attention-deficit hyperactivity disorder (ADHD) drugs and clinical characteristics of child and adolescent psychiatric outpatients prescribed multiple ADHD drugs in Japan**

Dear editor and reviewers:

Thank you for reviewing our manuscript and providing us your valuable advice despite your busy schedules.

We addressed all points raised by the academic editor and reviewers. In addition, to better facilitate the readers’ understanding, we modified some parts of the manuscript that were not pointed out by the reviewers. After these changes, scientific and academic editing services were used to re-edit the revised version of our manuscript before resubmission.

We would appreciate it if you could let us know again whether there are still parts that need further explanation or should be corrected.

Sincerely,

Masahide Usami

Department of Child and Adolescent Psychiatry, Kohnodai Hospital, National Center for Global Health and Medicine, 1-7-1 Kohnodai, Ichikawa, Chiba, 272-8516, Japan

Email: usami.masahide@hospk.ncgm.go.jp

Tel: +81-47-352-3501

**Response to Journal Requirements**

1. Please ensure that your manuscript meets PLOS ONE's style requirements, including those for file naming.

Following the journal requirements, we have revised our manuscript. We revised Level 1 headings for all major sections to bold type and 18-pt font. We revised Level 2 headings for subsections of major sections to bold type and 16-pt font. After making sure that there are no typos or omissions, the paper is finally sent for English editing.

**Response to Journal requirements**

2. In your Data Availability statement, you have not specified where the minimal data set underlying the results described in your manuscript can be found.

Accordingly, “Study design and setting” in our manuscript have been revised. The data cannot be publicly deposited in the supporting information due to patient privacy. Data are available from the Ethical Committee of the National Center for Global Health and Medicine for researchers who meet the criteria for access to confidential data.

Manuscript Page 6

**Methods**

**Study Design and Setting**

The purpose, methods, and analyses of the study and details of how to refuse participation were posted in the hospital’s outpatient clinic. In addition, the study data were anonymized because patient correspondence was unnecessary throughout the study period.

REVISED

**Methods**

**Study Design and Setting**

The purpose, methods, and analyses of the study and details of how to refuse participation were posted in the hospital’s outpatient clinic. In addition, the study data were anonymized because patient correspondence was unnecessary throughout the study period. Information from this study may contain potentially identifiable patient information, and data sharing is restricted by the Ethical Committee of the National Center for Global Health and Medicine based on the Ethical Guidelines for Medical and Health Research Involving Human Subjects of Japan. However, the data could be accessed by contacting the Ethical Committee of the National Center for Global Health and Medicine through the email address rinrijm@hosp.ncgm.go.jp.

**Response to Journal Requirements**

3. Please include a separate caption for each figure in your manuscript.

We inserted each figure caption directly after the paragraph in which they are first cited. We replaced TIFF files to fit the style of PLOS ONE (the content itself was not changed).

**Response to Reviewer #1**

I have a few questions. #1 The authors stated that the clinical characteristics associated with the use of three drugs were severe ADHD symptoms, autistic characteristics, or a tendency of child-to-parent violence at the initial consultation. How do you assess these autistic characteristics, or a tendency of child-to-parent violence? I think that these symptoms might be caused by the symptoms of Post Traumatic Stress Disorder. #2 Related to #1, How do you assess the abuse types?

As per Reviewer #1’s comments, we added a description in the Recruitment and participants. Readers’ misunderstandings or questions can be avoided by explaining in detail what we have conducted at our facility.

Manuscript Page 6

**Methods** Recruitment and participants

Psychologists and psychiatrists together established the initial interview forms, which included demographic and clinical characteristics. TABS and ADHD-RS were then constructed. Psychiatrists specializing in child and adolescent psychiatry evaluated clinical findings diagnosed all patients according to the Diagnostic and Statistical Manual of Mental Disorders, Fifth Edition [1]. Patients with moderate-to-severe intellectual disability according to the DSM-5, organic brain disease, drug-induced psychiatric disease, traumatic brain injury, and genetic syndromes were referred to other medical institutions and were excluded from this study.

In this study, child-to-parent violence meant physical violence against parents living together or damage to property. We considered patients as having “antisocial behavior” when patients engaged in illegal activities, such as smoking, drinking, illegal drug use, shoplifting, or causing injury to others. In Japan, smoking and drinking are legal after the age of 20 years. “Abuse experience” included sexual abuse, physical abuse, psychological abuse, and neglect. Refusal to attend school was defined as absence from school for ≥30 days owing to any psychological, emotional, physical, or social reasons [16].

REVISED

Psychologists and psychiatrists together established the initial interview forms, which included demographic and clinical characteristics. Consultation included the developmental history from caretakers, child observations, and school record reviews. TABS and ADHD-RS were then constructed. Psychiatrists specializing in child and adolescent psychiatry evaluated clinical findings, including psychological rating scales and diagnosed all patients according to the Diagnostic and Statistical Manual of Mental Disorders, Fifth Edition [1]. Meetings to discuss the psychopathology, symptoms, diagnosis, and possibility of abuse were conducted as needed. There were few patients ~~Patients~~ with moderate-to-severe intellectual disability according to the DSM-5, organic brain disease, drug-induced psychiatric disease, traumatic brain injury, and genetic syndromes because they were referred to other medical institutions when they call for an appointment in ~~and were excluded from~~ this study.

In this study, ~~child~~ the TABS was filled out by caretakers and then psychiatrists checked the items in a medical interview. The TABS score indicated the strength of autistic characteristics, and the ADHS-RS indicated the strength of ADHD symptoms as well. Child-to-parent violence meant physical violence against parents living together or damage to property. The in-charge psychiatrists interviewed patients and parents individually, as needed, to evaluate for child-to-parent violence. After hearing both sides of the story, psychiatrists decided whether the patients’ behavior was indicative of child-to-parent violence. We considered patients as having “antisocial behavior” when patients engaged in illegal activities, such as smoking, drinking, illegal drug use, shoplifting, or causing injury to others. In Japan, smoking and drinking are legal after the age of 20 years. In many cases, the evaluation of antisocial behavior would be based on information from parents. “Abuse experience” included sexual abuse, physical abuse, psychological abuse, and neglect. Our department worked with local child protection services. Hence, information about abused children was often available before the initial consultation. In addition, the in-charge psychiatrists interviewed parents and patients separately to identify abused children as needed. These efforts were made to immediately identify abused children and to prevent missed abused children. Refusal to attend school was defined as absence from school for ≥30 days owing to any psychological, emotional, physical, or social reasons [16].

**Response to Reviewer #2**

1) This study is done only in medical facility. Then, the result only reflects prescription pattern of small number of psychiatrists.

2) Medical information (such as the history of being abused) are based on only medical records.

Based on Reviewer #2’s comments, we have added a description to the Study limitations section as per the point made on the weakness of a retrospective study.

Manuscript Page 12

**Study Limitations**

This study has some limitations that should be considered. First, a selection bias may exist owing to several reasons. We did not track drug information before our consultation; therefore, patients in the control group may have been prescribed multiple ADHD drugs. Furthermore, this study did not represent the general situation associated with use multiple ADHD drugs among child and adolescent psychiatric patients because it was conducted in a single district. Second, this study may have been affected by information bias owing to several reasons. Information on the dosage and side effects of each ADHD drug, in what order and for how long it was prescribed, was not evaluated. Distinguishing whether ADHD drugs were used simultaneously or sequentially would reveal more acute clinical characteristics of patients who are resistant to ADHD drugs. In addition, this study did not include information on factors other than developmental characteristics and abuse, such as depression, and use of atypical antipsychotics for autistic characteristics. Therefore, this study only provides a partial view of the clinical characteristics of the patients experienced multiple ADHD drugs. Finally, the results of this study confirmed the association between use of three drugs and clinical characteristics, but not causality.

REVISED

This study has some limitations that should be considered. First, a selection bias may exist owing to several reasons. We did not track drug information before our consultation; therefore, patients in the control group may have been prescribed multiple ADHD medications ~~drugs~~. Furthermore, this study did not represent the general situation associated with use multiple ADHD medications ~~drugs~~ among child and adolescent psychiatric patients because it was conducted in a single district by a small number of psychiatrists. Second, this study may have been affected by information bias owing to several reasons. Information on a diagnosis was established after the first consultation. However, a diagnosis may change, and additional information may be included after further examinations. In total, 878 participants had a diagnosis of ADHD, but comorbidities such as ASD were not examined. Information on the dosage and side effects of each ADHD medication ~~drug~~, in what order and for how long it was prescribed, was not evaluated between two groups. Distinguishing whether ADHD medications ~~drugs~~ were used simultaneously or sequentially would reveal more acute clinical characteristics of patients who are resistant to ADHD medications ~~drugs~~. Information on abuse history was established after the first consultation. However, additional information regarding abuse history may be included after further examinations. Inaccuracies were present in the detection of abuse history because many abuses were not immediately visible. In other words, we might have missed an abuse history. In addition, this study did not include information on factors other than developmental characteristics and abuse, such as depression, and use of atypical antipsychotics for autistic characteristics. Because we wanted to make the study more relevant to actual clinical situations　that fits the clinician's sense, we did not divide the transition period in May 2017 when guanfacine became available. The results might change if the study was conducted after guanfacine is widely available. Therefore, this study only provides a partial view of the clinical characteristics of the patients received ~~experienced~~ multiple ADHD medications ~~drugs~~. Finally, the results of this study confirmed the association between use of three medications ~~drugs~~ and clinical characteristics, but not causality.

3) Samples are collected in 2015-2020. Among this period, some new anti-ADHD agents are marketed.This changed the prescription status. Authors must address why authors can collapse these data into the same datasets.

Accordingly, we have revised the content of the Study limitations section.

Manuscript Page 12

**Study Limitations**

In addition, this study did not include information on factors other than developmental characteristics and abuse, such as depression, and use of atypical antipsychotics for autistic characteristics. Therefore, this study only provides a partial view of the clinical characteristics of the patients experienced multiple ADHD drugs. Finally, the results of this study confirmed the association between use of three drugs and clinical characteristics, but not causality.

REVISED

In addition, this study did not include information on factors other than developmental characteristics and abuse, such as depression, and use of atypical antipsychotics for autistic characteristics. Because we wanted to make the study more relevant to actual clinical situations that fits the clinician's sense, we did not divide the transition period in May 2017 when guanfacine became available. The results might change if the study was conducted after guanfacine is widely available. Therefore, this study only provides a partial view of the clinical characteristics of the patients ~~experienced~~ received multiple ADHD ~~drugs~~ medications. Finally, the results of this study confirmed the association between use of three ~~drugs~~ medications and clinical characteristics, but not causality.

The PDF of Registry Study of this article is written in Japanese. It seems an opt-out.

We absolutely agree with your point. Therefore, we revised the link in the Methods section.

Manuscript Page 5

**Methods**

**Study design and setting**

This study used data from the Registry Study of Child and Adolescent Mental Health in Japan (http://www.ncgmkohnodai.go.jp/subject/100/200/opt10018111401.pdf). The study participants included patients who visited the Department of Child and Adolescent Psychiatry, Kohnodai Hospital, National Center for Global Health and Medicine between April 2015 and March 2020. We investigated patients who experienced OROS methylphenidate, atomoxetine, or guanfacine.

REVISED

This study used data from the Registry Study of Child and Adolescent Mental Health in Japan (http://www.ncgmkohnodai.go.jp/subject/100/optout.html~~http://www.ncgmkohnodai.go.jp/subject/100/200/opt10018111401.pdf~~). The study participants included patients who visited the Department of Child and Adolescent Psychiatry, Kohnodai Hospital, National Center for Global Health and Medicine between April 2015 and March 2020. We investigated patients with ADHD who ~~experienced~~ received OROS methylphenidate, atomoxetine, or guanfacine.

**Response to Reviewer #3**

1. Please reconsider the title of the manuscript, since it is somehow ambiguous.

The title has been revised as the comments of Reviewer #3.

**Title**

Status of attention-deficit hyperactivity disorder (ADHD) drugs and clinical characteristics of child and adolescent psychiatric outpatients prescribed multiple ADHD drugs in Japan

REVISED

Current use ~~Status~~ of attention-deficit hyperactivity disorder (ADHD) ~~drugs~~ medications and clinical characteristics of child and adolescent psychiatric outpatients prescribed multiple ADHD ~~drugs~~ medications in Japan

2. In the Abstract at the Conclusion section, you stated that your findings highlighted the approach to prevent multiple medications in children with ADHD. I am afraid that while you talked about the characteristics and possible reasons behind the multiple medications, I could not find notions regarding the approach/ways to avoid the multiple medications in the Discussion.

Accordingly, we revised the Conclusion section of the Abstract. “Highlight” is an overstatement; thus, we believe the more appropriate expression is “suggest.”

**Abstract Conclusions**

Our findings highlight the approach to prevent the use of multiple ADHD drugs. A prospective study to investigate the causality between prescribing status and clinical characteristics is warranted.

REVISED

Our findings suggest ~~highlight~~ the approach to prevent the use of multiple ADHD ~~drugs~~ medications. A prospective study to investigate the causality between prescribing status and clinical characteristics is warranted.

3. The term prescription status sounds confusing. It refers to a status of a patient prescription (either active, pending, or cancelled etc.). Similarly, prescribing status means if a physician or pharmacist is currently prescribing medications or not. I think you are discussing the current medication use and it would be better to re-check the use of those terms with the English editing service. Moreover, could you also check the term patients who experienced multiple ADHD drugs? I reckon patients who were multiply medicated or who received multiple ADHD medications or patients who were treated with all three ADHD medications is more appropriate. The word experience highlights the meaning of subjective experience of a person taking medication.

As suggested, we changed some words that might cause confusion to the reader. Specifically, we changed "prescription status" to "the current medication use" and "who experienced multiple ADHD drugs" to "who received multiple ADHD medications. Please refer to the manuscript at their several instances. Thank you for your pertinent and thoughtful comments.

4. In the final paragraph of the Introduction, you state that although it is not appropriate to simultaneously prescribe all three ADHD medications, a sequential trial of those is reasonable. You had better clarify why it is not appropriate to simultaneously prescribe all three ADHD medications.

Accordingly, we added the reason to the Introduction section.

Manuscript Page 5

**Introduction**

This study investigated the current medication use of ADHD medications and the characteristics of patients who received multiple ADHD medications in a clinical setting in Japan. It is inappropriate to simultaneously use all three medications. However, it seems reasonable to use the three medications sequentially in certain conditions. We hypothesized that patients who received multiple ADHD medications tend to have more autistic symptoms than those who were not.

REVISED

This study investigated the current ~~medication~~ use of ADHD medications and ~~the~~ characteristics of patients who received multiple ADHD medications in a clinical setting in Japan. It is inappropriate to simultaneously use all three medications for the potential risks of side effects. However, it seems reasonable to use ~~the~~ three medications sequentially in certain conditions. The comorbidity of ADHD and autism spectrum disorder (ASD) is approximately 30%–50% [9, 10]. Methylphenidate was effective in treating hyperactivity associated with ASD, but the magnitude of the response was less than that noted in typical ADHD [11]. We hypothesized that patients who received multiple ADHD medications tend to have more autistic symptoms than those who did ~~were~~ not. ADHD frequently occurs simultaneously with other neurodevelopmental conditions, such as ASD.

5. In the final paragraph of the Introduction, it is better to present the argument of ADHD-ASD co-occurrence/ADHD medication use in ASD (i.e. reasons behind your hypothesis) before stating your hypothesis.

Accordingly, we revised the final paragraph of the Introduction section.

Manuscript Page 5

**Introduction**

We hypothesized that patients who received multiple ADHD medications tend to have more autistic symptoms than those who were not. ADHD frequently occurs simultaneously with other neurodevelopmental conditions, such as autism spectrum disorder (ASD). The comorbidity of ADHD and ASD is approximately 30%–50% [9, 10]. Methylphenidate was effective in treating hyperactivity associated with ASD, but the magnitude of the response was less than that noted in typical ADHD [11].

REVISED

The comorbidity of ADHD and ASD is approximately 30%–50% [9, 10]. Methylphenidate was effective in treating hyperactivity associated with ASD, but the magnitude of the response was less than that noted in typical ADHD [11]. We hypothesized that patients who received multiple ADHD medications tend to have more autistic symptoms than those who did ~~were~~ not. ADHD frequently occurs simultaneously with other neurodevelopmental conditions, such as autism spectrum disorder (ASD). ~~The comorbidity of ADHD and ASD is approximately 30%–50% [9, 10]. Methylphenidate was effective in treating hyperactivity associated with ASD, but the magnitude of the response was less than that noted in typical ADHD [11].~~

6. In the Method, the inclusion and exclusion criteria are not presented clearly enough. What was the targeted age range (the minimum age)? Did you include patients who were on any of the three ADHD medications continuously for more than 180 days regardless of their diagnoses? What were the diagnoses of 878 participants including comorbidity? As you mentioned in the Introduction, ADHD and ASD co-occur to a significant degree. Did you exclude or include (for the whole sample and the case and control groups) individuals with ASD and if so how did you make the diagnosis (like using the ADOS-2 or ADI-R)?

Further, could you describe what you mean by autistic characteristics (Does this mean patients presenting some ASD symptoms but without ASD diagnosis or include those with ASD diagnosis)? In addition, how did you diagnose ADHD (I understand that expert psychiatrists and psychologists in the field established interview forms and made diagnoses but there are no references to the intake of developmental history from parents, observation of a child, or reviewing of school records etc.)? More details for the description of the diagnostic process are preferable.

Accordingly, we revised the Method and Study limitations sections.

Manuscript Page 5

**Method**

**Study design and setting**

This study used data from the Registry Study of Child and Adolescent Mental Health in Japan (http://www.ncgmkohnodai.go.jp/subject/100/optout.html). The study participants included patients who visited the Department of Child and Adolescent Psychiatry, Kohnodai Hospital, National Center for Global Health and Medicine between April 2015 and March 2020. We investigated patients who received OROS methylphenidate, atomoxetine, or guanfacine. We examined the proportion of patients who had been prescribed for ≥180 days to determine the response rate of each drug treatment based on previous studies [12, 13]. Furthermore, a retrospective case–control design was used to evaluate the clinical characteristics of patients resistant to ADHD medications. Patients who received all three medications, including OROS methylphenidate, atomoxetine, and guanfacine, were defined as the case group. The patients in the case group had ADHD as their diagnosis. Conversely, randomly sampled sex- and age-matched patients diagnosed with ADHD at initial consultation during the same period were defined as the control group. The purpose of this study was to investigate the differences between patients diagnosed with ADHD and patients who received multiple ADHD medications. In line with the case–control design, we compared data for child-to-parent violence, antisocial behavior, suicide attempt or self-harm, abuse history, refusal to attend school, and two psychological rating scales, the ADHD-Rating Scale (ADHD-RS) [14] and the Tokyo Autistic Behavior Scale (TABS) [15].

REVISED

This study used data from the Registry Study of Child and Adolescent Mental Health in Japan (http://www.ncgmkohnodai.go.jp/subject/100/optout.html). The study participants included patients who visited the Department of Child and Adolescent Psychiatry, Kohnodai Hospital, National Center for Global Health and Medicine between April 2015 and March 2020. We investigated patients with ADHD who received OROS methylphenidate, atomoxetine, or guanfacine. In Japan, only patients with ADHD diagnosis can receive ADHD medication, regardless of whether it is a primary or secondary diagnosis, and with or without comorbidities. We examined the proportion of patients who had been prescribed for ≥180 days to determine the response rate of each drug treatment based on previous studies [12, 13]. Furthermore, a retrospective case–control design was used to evaluate the clinical characteristics of patients resistant to ADHD medications. Patients who received all three medications, including OROS methylphenidate, atomoxetine, and guanfacine, were defined as the case group. The patients in the case group had ADHD as their diagnosis. Conversely, randomly sampled sex- and age-matched patients diagnosed with ADHD at initial consultation during the same period were defined as the control group. ~~The purpose~~ Therefore, of this study ~~was~~ aimed to investigate ~~the~~ differences between patients diagnosed with ADHD and patients who received multiple ADHD medications. For the two-group comparison, we compared ADHD diagnosed children with a use of no more than 2 ADHD drugs (i.e. tried zero or one or two drugs but not all three) versus ADHD diagnosed children who were treated with all three ADHD medications. In line with the case–control design, we compared data for child-to-parent violence, antisocial behavior, suicide attempt or self-harm, abuse history, refusal to attend school, and two psychological rating scales, the ADHD-Rating Scale (ADHD-RS) [14] and the Tokyo Autistic Behavior Scale (TABS) [15].

Manuscript Page 7

**Method**

**Recruitment and participants**

This study included patients aged ≤15 years at the first consultation who visited our department between April 2015 and March 2020. Most participants resided in Ichikawa City and the surrounding areas, which has a population of approximately 492,393 (as calculated in August 2020).

Psychologists and psychiatrists together established the initial interview forms, which included demographic and clinical characteristics. TABS and ADHD-RS were then constructed. Psychiatrists specializing in child and adolescent psychiatry evaluated clinical findings which included psychological rating scales and diagnosed all patients according to the Diagnostic and Statistical Manual of Mental Disorders, Fifth Edition [1]. Meetings to discuss the psychopathology, the symptoms, diagnosis and the possibility of abuse were conducted as needed. Patients with moderate-to-severe intellectual disability according to the DSM-5, organic brain disease, drug-induced psychiatric disease, traumatic brain injury, and genetic syndromes were referred to other medical institutions and were excluded from this study.

In this study, child-to-parent violence meant physical violence against parents living together or damage to property. The in-charge psychiatrists interviewed patients and parents individually, as needed, to evaluate for child-to-parent violence. After hearing both sides of the story, the psychiatrists decided whether the patients’ behavior was indicative of child-to-parent violence. We considered patients as having “antisocial behavior” when patients engaged in illegal activities, such as smoking, drinking, illegal drug use, shoplifting, or causing injury to others. In Japan, smoking and drinking are legal after the age of 20 years. “Abuse experience” included sexual abuse, physical abuse, psychological abuse, and neglect. Our department worked with local child protection services. Hence, information about abused children were often available before the initial consultation. In addition, the in-charge psychiatrists interviewed parents and patients separately to identify abused children as needed. These efforts were made to immediately identify abused children and to prevent missed abused children. Refusal to attend school was defined as absence from school for ≥30 days owing to any psychological, emotional, physical, or social reasons [16].

REVISED

This study included patients aged ≤15 years at the first consultation who visited our department between April 2015 and March 2020. Patients aged <1 year also came to the clinic, but teenagers made up majority of patients. Most participants resided in Ichikawa City and the surrounding areas, which has a population of approximately 492,393 (as calculated in August 2020).

Psychologists and psychiatrists together established the initial interview forms, which included demographic and clinical characteristics. Consultation included the developmental history from caretakers, child observations, and school record reviews. TABS and ADHD-RS were then constructed. Psychiatrists specializing in child and adolescent psychiatry evaluated clinical findings, ~~which included~~ including psychological rating scales and diagnosed all patients according to the Diagnostic and Statistical Manual of Mental Disorders, Fifth Edition [1]. Meetings to discuss the psychopathology, ~~the~~ symptoms, diagnosis, and ~~the~~ possibility of abuse were conducted as needed. ~~Patients~~ There were few patients with moderate-to-severe intellectual disability according to the DSM-5, organic brain disease, drug-induced psychiatric disease, traumatic brain injury, and genetic syndromes were referred to other medical institutions ~~and were excluded from~~ when they call for an appointment in this study.

In this study, the TABS was filled out by the caretakers and then psychiatrists checked the items in a medical interview. The TABS score indicated the strength of autistic characteristics, and the ADHS-RS indicated the strength of ADHD symptoms as well. C~~c~~hild-to-parent violence meant physical violence against parents living together or damage to property. The in-charge psychiatrists interviewed patients and parents individually, as needed, to evaluate for child-to-parent violence. After hearing both sides of the story, ~~the~~ psychiatrists decided whether the patients’ behavior was indicative of child-to-parent violence. We considered patients as having “antisocial behavior” when patients engaged in illegal activities, such as smoking, drinking, illegal drug use, shoplifting, or causing injury to others. In Japan, smoking and drinking are legal after the age of 20 years. In many cases, the evaluation of antisocial behavior would be based on information from parents. “Abuse experience” included sexual abuse, physical abuse, psychological abuse, and neglect. Our department worked with local child protection services. Hence, information about abused children ~~were~~ was often available before the initial consultation. In addition, the in-charge psychiatrists interviewed parents and patients separately to identify abused children as needed. These efforts were made to immediately identify abused children and to prevent missed abused children. Refusal to attend school was defined as absence from school for ≥30 days owing to any psychological, emotional, physical, or social reasons [16].

Manuscript Page 12

**Study Limitations**

This study has some limitations that should be considered. First, a selection bias may exist owing to several reasons. We did not track drug information before our consultation; therefore, patients in the control group may have been prescribed multiple ADHD medications. Furthermore, this study did not represent the general situation associated with use multiple ADHD medications among child and adolescent psychiatric patients because it was conducted in a single district by small number of psychiatrists. Second, this study may have been affected by information bias owing to several reasons. Information on the dosage and side effects of each ADHD medication, in what order and for how long it was prescribed, was not evaluated. Distinguishing whether ADHD medications were used simultaneously or sequentially would reveal more acute clinical characteristics of patients who are resistant to ADHD medications. Information on abuse history were established after the first consultation. However, additional information regarding abuse history may be included after further examinations. Inaccuracies were present in the detection of abuse history because many of the abuses were not immediately visible. In other words, we might have missed an abuse history. In addition, this study did not include information on factors other than developmental characteristics and abuse, such as depression, and use of atypical antipsychotics for autistic characteristics. Because we wanted to make the study more relevant to actual clinical situations, we did not deliberately divide the transition period when guanfacine became available. The results might change if the study was conducted after guanfacine is widely available. Therefore, this study only provides a partial view of the clinical characteristics of the patients received multiple ADHD medications. Finally, the results of this study confirmed the association between use of three medications and clinical characteristics, but not causality.

REVISED

This study has some limitations that should be considered. First, a selection bias may exist owing to several reasons. We did not track drug information before our consultation; therefore, patients in the control group may have been prescribed multiple ADHD medications. Furthermore, this study did not represent the general situation associated with use multiple ADHD medications among child and adolescent psychiatric patients because it was conducted in a single district by a small number of psychiatrists. Second, this study may have been affected by information bias owing to several reasons. Information on a diagnosis was established after the first consultation. However, a diagnosis may change and additional information may be included after further examinations. In total, 878 participants had a diagnosis of ADHD but comorbidities such as ASD were not examined. Information on the dosage and side effects of each ADHD medication, in what order and for how long it was prescribed, was not evaluated between two groups. Distinguishing whether ADHD medications were used simultaneously or sequentially would reveal more acute clinical characteristics of patients who are resistant to ADHD medications. Information on abuse history was ~~were~~ established after the first consultation. However, additional information regarding abuse history may be included after further examinations. Inaccuracies were present in the detection of abuse history because many ~~of the~~ abuses were not immediately visible. In other words, we might have missed an abuse history. In addition, this study did not include information on factors other than developmental characteristics and abuse, such as depression, and use of atypical antipsychotics for autistic characteristics. Because we wanted to make the study more relevant to actual clinical situations that fits the clinician’s sense, we did not ~~deliberately~~ divide the transition period in May 2017 when guanfacine became available. The results might change if the study was conducted after guanfacine is widely available. Therefore, this study only provides a partial view of the clinical characteristics of the patients received multiple ADHD medications. Finally, the results of this study confirmed the association between use of three medications and clinical characteristics, but not causality.

7. You should describe the medication dosage of the two groups.

As suggested, we added a few explanations at the Study limitations section. Unfortunately, we have not been able to determine the dosage.

Manuscript Page 12

**Study Limitations**

Information on the dosage and side effects of each ADHD medication, in what order and for how long it was prescribed, was not evaluated. Distinguishing whether ADHD medications were used simultaneously or sequentially would reveal more acute clinical characteristics of patients who are resistant to ADHD medications.

REVISED

Information on the dosage and side effects of each ADHD medication, in what order and for how long it was prescribed, was not evaluated between two groups. Distinguishing whether ADHD medications were used simultaneously or sequentially would reveal more acute clinical characteristics of patients who are resistant to ADHD medications.

8. For the two-group comparison, I would like to clarify that it is comparing ADHD diagnosed children with a use of no more than 2 ADHD drugs (i.e. tried zero or one or two drugs but not all three) versus ADHD diagnosed children who were treated with all three ADHD medications. Were they diagnosed only with ADHD without any comorbidity including ASD/depression/anxiety disorders?

Accordingly, we revised the Methods section. As we mentioned in the limitation section, participants had a diagnosis of ADHD, but comorbidities such as ASD, depression, or anxiety disorders were not examined.

Manuscript Page 6

**Methods**

**Study design and setting**

The purpose of this study was to investigate the differences between patients diagnosed with ADHD and patients who received multiple ADHD medications. In line with the case–control design, we compared data for child-to-parent violence, antisocial behavior, suicide attempt or self-harm, abuse history, refusal to attend school, and two psychological rating scales, the ADHD-Rating Scale (ADHD-RS) [14] and the Tokyo Autistic Behavior Scale (TABS) [15].

REVISED

~~The purpose~~ Therefore, of this study ~~was~~ aimed to investigate ~~the~~ differences between patients diagnosed with ADHD and patients who received multiple ADHD medications. For the two-group comparison, we compared ADHD diagnosed children with a use of no more than 2 ADHD drugs (i.e., tried zero or one or two drugs but not all three) versus ADHD diagnosed children who were treated with all three ADHD medications. In line with the case–control design, we compared data for child-to-parent violence, antisocial behavior, suicide attempt or self-harm, abuse history, refusal to attend school, and two psychological rating scales, the ADHD-Rating Scale (ADHD-RS) [14] and the Tokyo Autistic Behavior Scale (TABS) [15].

9. For those who used more than 2 medications, did they switch medications due to insufficient treatment effects or was the switch due to adverse effects?

As we mentioned in the Study limitation section, we did not investigate whether patients switched drugs because of insufficient therapeutic effects or because of side effects. This is a future challenge that should be considered.

10. You excluded patients with moderate to severe intellectual disability. How did you assess the intelligence?

We did not perform intelligence tests on all of our patients. However, when patients call for appointments, we check their approximate intelligence based on their academic performance, and a patient with more than moderate intellectual disabilities actually rarely comes to our clinic. We changed the content of Recruitment and participants section to an appropriate sentence.

Manuscript Page 7

**Methods**

**Recruitment and participants**

Patients with moderate-to-severe intellectual disability according to the DSM-5, organic brain disease, drug-induced psychiatric disease, traumatic brain injury, and genetic syndromes were referred to other medical institutions and were excluded from this study.

REVISED

There were few p~~P~~atients with moderate-to-severe intellectual disability according to the DSM-5, organic brain disease, drug-induced psychiatric disease, traumatic brain injury, and genetic syndromes because they were referred to other medical institutions when they call for an appointment in ~~and were excluded from~~ this study.

11. How did you assess the child-to-parent violence, anti-social behaviors, or abuse? Was it based on a parent report or asked to a patient in the clinician interview? It is possible that reports on these sensitive episodes, especially abuse, are different between those made by parents and those by children and I am wondering how and from whom you extracted this information.

Based on Reviewer #3’s comment, we have added a description in the Recruitment and participants.

Manuscript Page 6

**Methods** Recruitment and participants

In this study, child-to-parent violence meant physical violence against parents living together or damage to property. We considered patients as having “antisocial behavior” when patients engaged in illegal activities, such as smoking, drinking, illegal drug use, shoplifting, or causing injury to others. In Japan, smoking and drinking are legal after the age of 20 years. “Abuse experience” included sexual abuse, physical abuse, psychological abuse, and neglect. Refusal to attend school was defined as absence from school for ≥30 days owing to any psychological, emotional, physical, or social reasons [16].

REVISED

In this study, child-to-parent violence meant physical violence against parents living together or damage to property. The in-charge psychiatrists interviewed patients and parents individually, as needed, to evaluate for child-to-parent violence. After hearing both sides of the story, psychiatrists decided whether the patients’ behavior was indicative of child-to-parent violence. We considered patients as having “antisocial behavior” when patients engaged in illegal activities, such as smoking, drinking, illegal drug use, shoplifting, or causing injury to others. In Japan, smoking and drinking are legal after the age of 20 years. In many cases, the evaluation of antisocial behavior would be based on information from parents. “Abuse experience” included sexual abuse, physical abuse, psychological abuse, and neglect. Our department worked with local child protection services. Hence, information about abused children was often available before the initial consultation. In addition, the in-charge psychiatrists interviewed parents and patients separately to identify abused children as needed. These efforts were made to immediately identify abused children and to prevent missed abused children. Refusal to attend school was defined as absence from school for ≥30 days owing to any psychological, emotional, physical, or social reasons [16].

12. Is TABS applied to a general population to assess ASD symptoms like the Autism Spectrum Quotient or only to assess current ASD symptoms in those diagnosed with ASD? Is there any cut-off? It may be helpful to explain it in detail as it is used only in Japan.

TABS is applied to both the general population and those diagnosed with ASD to assess ASD symptoms. There is no cut-off. Autistic features change over the course of development; thus, a clinician should identify behaviors in early infancy. TABS is a psychological scale to assist in the diagnosis of ASD by assessing current and past behaviors.

Manuscript Page 8

**Measurements**

TABS is a tool used by a child’s caretaker to assess the behavior of children with autism. It comprises 39 items that are provisionally grouped into four areas: interpersonal–social relationship, language–communication, habit–mannerism, and others. Each item is scored from 0 to 2 and has a highest score of 78. Higher scores indicate strong current autistic characteristics [15].

REVISED

TABS is a tool used by a child’s caretaker to assess the current and past behaviors of children with autism. Autistic features change over the course of development; thus, a clinician should know their behaviors in early infancy. It comprises 39 items that are provisionally grouped into four areas: interpersonal–social relationship, language–communication, habit–mannerism, and others. Each item is scored from 0 to 2 and has a highest score of 78. It is applied to both the general population and those diagnosed with ASD. It has no cut-off point. Higher scores indicate strong current autistic characteristics [15].

13. In the Method about the statistical analysis, the sentences for deciding on the explanatory variables for the univariate and multivariate analyses are not clear.

Accordingly, we added a few explanations in the Statistical analysis section.

Multivariate regression analysis was performed to determine variables that were independently associated with patients who received three ADHD medications (OROS methylphenidate, atomoxetine, and guanfacine). Odds ratios and 95% confidence interval were calculated using univariate and multivariate logistic regression models (Tables 2 and 3).

REVISED

Multivariate regression analysis was performed to determine variables that were independently associated with patients who received three ADHD medications (OROS methylphenidate, atomoxetine, and guanfacine). Possible confounding factors selected from previous studies and medical viewpoints, as described in the Introduction section, were included for multivariate logistic analysis. Odds ratios and 95% confidence interval were calculated using univariate and multivariate logistic regression models (Tables 2 and 3).

14. In the Result, the age ranged from 10 to 15 years old (written below Table1), however the mean age is 8.55. How is this possible?

As suggested, we revised the additional information in the table and apologize for this mistake.

Manuscript Page 10

**Table 1**

Score ranges: Age, 10–15 years; TABS, 0–78 points; ADHD-RS, 0–54 points

REVISED

Score ranges: Age, ≤~~10–~~15 years; TABS, 0–78 points; ADHD-RS, 0–54 points

15. In the Discussion Page 9 Line 19, approximately is not spelled right.

Accordingly, we revised misspelled word in the Discussion section.

approxmately→approximately

16. In the Discussion Page 10 Line 12-18, you talk about the relationship between depression and violence and its possibility underlying the insufficient response to a single ADHD medication. I understand the importance but did you assess the depression/anxiety in the study or did you include those with ADHD comorbid with depression? Did any of the participants used anti-depressives along with ADHD medications? It seems a little leap to state the association with depression without mentioning such information.

As per Reviewer #3’s comments, we revised the Discussion section. As indicated, this was a leap of faith in the Discussion section.

Regarding child-to-parent violence, ADHD medications might be prescribed to reduce impulsivity as much as possible because reactive-impulsive violence was related to ADHD psychopathology [31]. Cohort studies reported that depression in young people was associated with subsequent violent outcomes [32]. Furthermore, a systematic review and meta-analysis reported that child maltreatment or exposure to domestic violence was associated with an increased risk of interpersonal violence [33]. This finding indicates that ADHD symptoms among patients with a tendency of child-to-parent violence requires approaches other than ADHD medications, such as treatment for depression and/or abuse.

REVISED

Regarding child-to-parent violence, ADHD medications might be prescribed to reduce impulsivity as much as possible because reactive-impulsive violence was related to ADHD psychopathology [31]. Cohort studies reported that depression in young people was associated with subsequent violent outcomes [32]. Furthermore, a systematic review and meta-analysis reported that child maltreatment or exposure to domestic violence was associated with an increased risk of interpersonal violence [33]. This finding suggests the need for future research on the relationship between patients with child-to-parent violence who receive multiple medications and a history of depression and abuse. ~~This finding indicates that ADHD symptoms among patients with a tendency of child-to-parent violence requires approaches other than ADHD medications, such as treatment for depression and/or abuse.~~
